# Movements During Intended General Anesthesia and Psychologically Traumatic Accidental Awareness: Explanatory Role of the “Efference Copy Network”

**DOI:** 10.1213/ANE.0000000000007722

**Published:** 2025-09-05

**Authors:** Jaideep J. Pandit

**Affiliations:** From the 1 Nuffield Department of Anaesthetics, Oxford University Hospitals NHS Foundation Trust, Oxford, UK; 2 Nuffield Department of Clinical Neurosciences, University of Oxford, Oxford, UK.

Two key results of the fifth National Audit Project (NAP5) in the United Kingdom, the largest ever study into accidental awareness during general anesthesia (AAGA) were that (i) AAGA is virtually confined to when neuromuscular blockade, NMB, is used (1 in 8000 vs 1 in 136,000 with no NMB)^1^; (ii) psychological trauma after AAGA only occurs in patients administered NMBs.^2^ With AAGA, paralysis was more distressing than any feelings of pain: patients at least recognized pain, but the inability to move was something new, resulting in feelings of entombment and even later posttraumatic stress disorder.^2^

## THE SIGNIFICANCE OF MOVEMENTS DURING ANESTHESIA AND THEIR DIFFERENT FORMS

Normally when awake, movement is a natural, common, and often important response to an external stimulus. During anesthesia with no NMB, movements are also common with jerks of limbs or facial grimacing. These spontaneous movements, often contiguous with surgical stimulus, are interpreted as signs of “light” anesthesia and readily abolished by deepening hypnosis and/or analgesia. By “spontaneous” is meant here a movement in the absence of any command.

Quite separately are the experimental results from the “isolated forearm technique” (IFT). This was introduced by Tunstall in the 1970s to detect AAGA in the days before sophisticated brain monitoring.^3^ After inducing anesthesia, a pressure cuff is inflated to prevent blood flow to one forearm, after which NMB is administered to the contralateral arm. The cuff need not be permanently inflated as after NMB drug binding and elimination of any circulating NMB has occurred, nerve stimulator monitoring to each hand can confirm the paralysis in the rest of the body, except for the previously isolated arm.^4^ The patient thus retains motor capacity to signal any awareness during the procedure.

However, results using IFT require careful interpretation. A systematic review (published as part of a “pro-con” debate^5^ showed that IFT studies consistently report that patients do not move spontaneously, as if to alert caregivers of awareness; but instead they move their fingers to simple verbal commands.^6,7^ In a minority of IFT cases (11% as reported in the systematic review^5^), jerky movements not always contiguous with painful stimuli can be observed. Examples of such studies include trials reported by Russell, where anesthetic (inhalational^8^ and, separately, intravenous^9^) was titrated to achieve bispectral index (BIS) values 55 to 60 which, notwithstanding limitations of this monitoring technology, are considered in national guidance to be suitable depths of anesthesia for surgery.^10^

For anyone who has not seen an IFT patient respond to command, a publicly accessible video shows the motor response to verbal command, when before this there has been no spontaneous or voluntary movement (see https://www.youtube.com/watch?v=ZEAYsEbkJrw). While the details of the anesthetic in the video (dosing, use of opiates or epidural, etc) are unknown, this is a response that can be reproduced by any anesthesiologist, anywhere, using anesthetic techniques and dosing reported elsewhere.^4,8,9^ It is important to stress that the only value—the very essence or *raison d’etre*—of the IFT is in the giving of the verbal command. The IFT has never been practised as a passive construct, wherein the patient is expected to move voluntarily to signify their wakefulness. Rather, the IFT permits anesthetic titration to low concentrations: at the point of movement to verbal command, the dose is increased to prevent further movement. It can therefore be argued that an IFT response to verbal command occurs only when anesthetic concentrations are “unacceptably” low (regardless of BIS levels being within the acceptable range, as in Russell’s studies cited above^8,9^). However, these low anesthetic concentrations do not correspond to the patient moving spontaneously, to signify wakefulness, but only to the verbal command. Moreover, the emphasis is that the real clinical utility of IFT lies in the “no-response.” In other words, regardless of how low is the anesthetic concentration (even if near-zero) a patient undergoing surgery in the IFT construct who neither moves spontaneously nor to repeated verbal command can be assuredly regarded as being adequately anesthetised. This cannot be said of any other “depth of anesthesia” monitor: eg, even very low values for processed EEG monitors can be misleading and patients have been accidentally aware, as reported in the NAP5 study.^1,2^

This pattern in IFT is the opposite of that seen in patients with no NMB, who clearly can move spontaneously during surgical stimulus, as described above, but as yet there is no report of these patients ever moving to verbal command. Two observational trials, including a total of 300 patients, have been unable to elicit movement to verbal command despite anesthetic concentrations being low and approximating the modest levels used in many IFT studies (BIS values 50–60). There is no report in the literature of any patient, anesthetised (no NMB), self-ventilating (eg, via a supraglottic airway), responding to verbal command, even when otherwise moving spontaneously to surgical stimulus.^11,12^

While anesthetic dosing is undoubtedly important to any interpretation, the question is not what is seen at relatively high concentrations (eg, ≥1 MAC; minimum alveolar concentration), which is not germane, because at these levels no responses (either spontaneous or response to command) are seen. Rather, the range of interest is the difference in responses between IFT and “no-NMB anesthesia” in the lower concentration range (eg, ≤1 MAC), and also in comparing IFT “partial paralysis” vs complete neuromuscular blockade.

**Figure 1.**
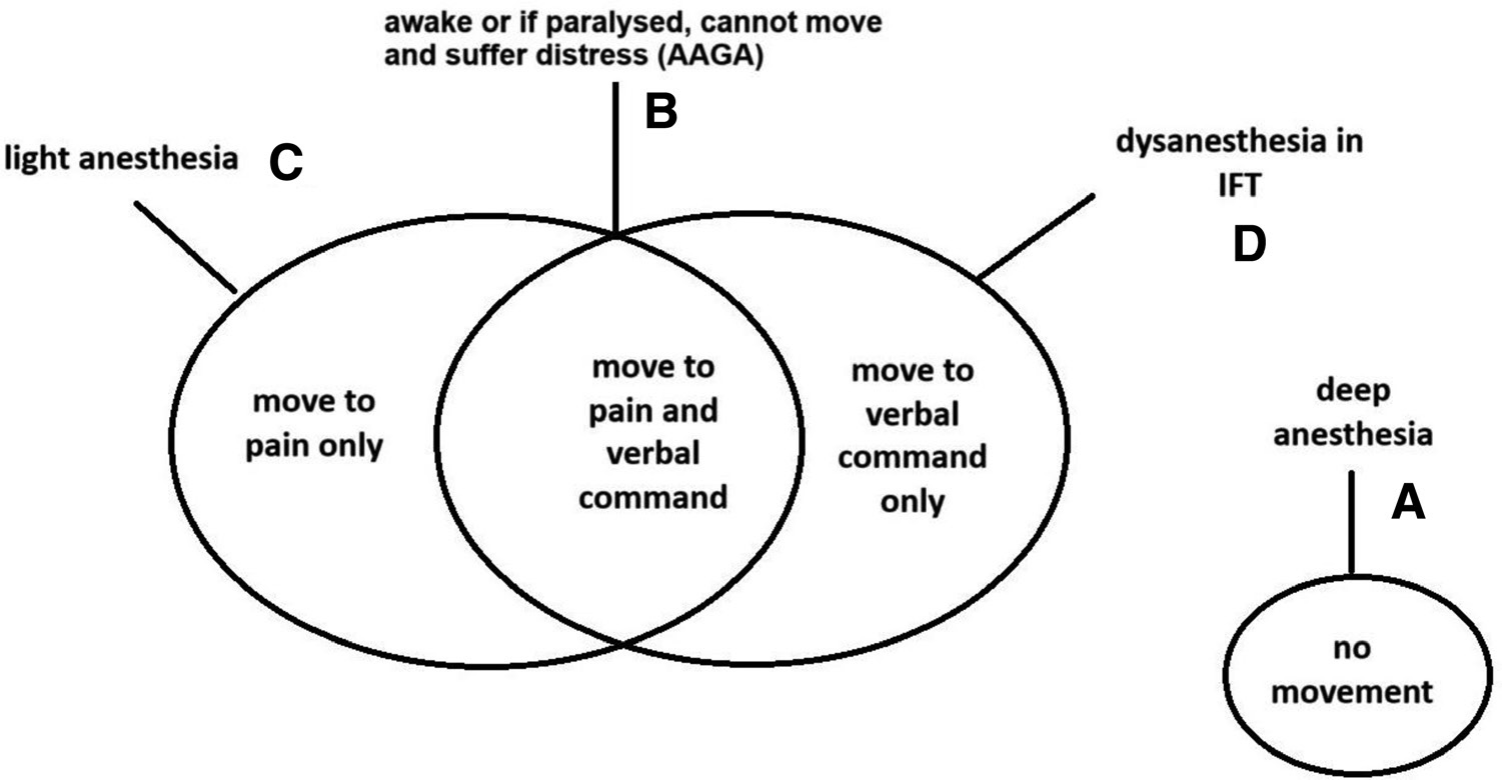
Summary of the responses seen in the 4 scenarios described in text. A, Deep anesthesia there is no movement. B, An awake patient will respond with movement to both pain and verbal command; but if paralyzed with NMB and with inadequate anesthesia may suffer traumatic awareness (AAGA) as they cannot move despite trying. C, In light anesthesia, there is no response to verbal command, but patients can move spontaneously to surgical stimulus. D, In the IFT construct at equivalently light anesthesia to (C), patients do not move to surgical stimulus, but do move to verbal command (a paradoxical pattern of response to (C) termed elsewhere as “dysanesthesia.”^6^)

The different combinations of anesthetic depths and intensities of neuromuscular blockade result in 4 broadly discrete patterns of response (Figure 1). First (A) is where anesthesia is sufficiently deep (eg, >1 MAC) that there is neither spontaneous movement nor movement to command. Second (B) is in intended anesthesia, which is in retrospect inadequate, with NMB: patients try to move but cannot, and can suffer traumatic AAGA (as shown by NAP5). Third (C) is in light anesthesia (eg, <1 MAC) with no NMB: patients can move spontaneously, but never to verbal command; AAGA is extremely rare and not traumatic. Finally (D) is the IFT construct where with light anesthesia patients can move to verbal command, but rarely spontaneously; there is little or no recall and no trauma.

It is important to emphasize that in this categorization of 4 patterns of response, no deeper meaning is ascribed to the movements in the sense of whether they are “voluntary” or signify “consciousness.” Moreover, they cannot, and should not, be mapped onto our different and more conventional understanding of MAC. Whereas MAC is an important tool to help normalize anesthetic concentration-response relationships across different agents at population level, especially in pharmacological investigation, in an individual patient whose response can only be binary (moving or not moving) it has less utility.^13^

IFT responses challenge our view on MAC. Leave an IFT patient undisturbed and they are “anesthetized” in the traditional MAC-concept if they do not move to surgical stimulus. Then, give them a verbal command and at exactly the same surgical stimulus, anesthetic concentration, opiate dose, epidural level, etc, they move in response to the instruction. They are no longer “anesthetised” in the MAC-framework. To adopt a phrase used elsewhere in the different context of the dynamics of emergence from anesthesia, but which is also applicable here, IFT patients can be like Schrodinger’s cat; both awake and anesthetised under the same conditions if—and only if—we insist on interpreting all observations within the MAC-framework.^14^ Since this feline analogy is interesting but leads nowhere, it is important to avoid interpreting these observations within the conventional framework of MAC.

A different theory is needed to explain these patterns, and thereby explain a question earlier posed^7^: “why is there never a response to command in unparalysed anesthetised patients?”

## THE EFFECT OF NMB ON AROUSAL

Central to developing any theory is to consider the potential influences of NMB on arousal. Traditionally, neuromuscular blockade was thought to contribute to anesthesia depth through “deafferentation.”^8^ The putative sedative effect of pure neuraxial anesthesia was used as evidence in support of deafferentation theory.^15^ Similarly, NMB-induced paralysis leading to proprioceptor inhibition was purported to reduce afferent traffic to the brain. Neuromuscular blockade was observed to decrease EEG power and shift it towards slower frequencies with increased periods of burst suppression and isoelectricity.^16^ The notion that NMBs reduce arousal was the basis of the historical “Liverpool technique” of anesthesia which relied predominantly on deep motor block and less on hypnotic agents.^17^

There were, however, several weaknesses of the deafferentation theory.^18,19^ One is that, unlike locoregional block, NMBs preserve other afferent traffic so should not significantly reduce overall feedback stimulation if only influencing proprioception. A second is that the NMB-induced reduction in high frequency EEG activity is possibly because this arises from muscle tone.^20^ Third is the evidence from studies in awake volunteers, that attempts to move in the face of global paralysis with NMB is distressing (arousing).^21–23^ This was associated with BIS values in these awake volunteers falling to as low as 44 (ie, the processed EEG of BIS value does not always correlate with the conscious experience).^22^ Third is the now overwhelming evidence from NAP5, that AAGA and distress occur almost exclusively when NMBs are used (and again, sometimes in context of low BIS values).^1,2^ The Liverpool technique is now discredited,^18,19^ and in contrast, NAP5 through its national guidance now recommends that if a patient moves during surgery the correct first response is to deepen anesthesia and/or analgesia, not just to intensify NMB.^1,2^

These results in fact suggest that NMBs increase arousal, and this alternative view is also supported by IFT results. Because formulating a coherent movement response to verbal command (as in scenario D; Figure 1) implies a higher degree of mental faculty than nonspecific spontaneous movement (as in scenario C Figure 1; anesthesia with no NMB), it follows that the partial paralysis in IFT (ie, one arm-only free of NMB) results in a higher level of cortical arousal than no paralysis. In turn, this partial paralysis of IFT is, however, clearly not as arousing as the full paralysis of complete NMB which is associated with traumatic AAGA, as IFT patients do not exhibit later anxiety, even if they have later recall of the commands. Although IFT patients are generally primed that during surgery they will be given commands to move their isolated hand as a check of awareness, they have no expectation of anesthesia being inadequate and their being actually awake, nor has the anesthesiologist deliberately used a technique to create awareness.

**Figure 2.**
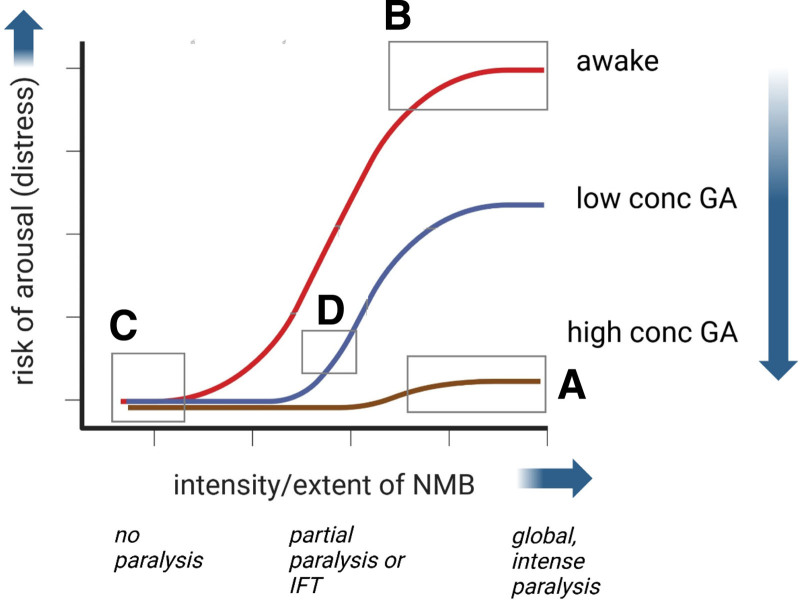
Interpretation of Figure 1 findings in context of interaction with anesthesia depth, cortical arousal and neuromuscular blockade (NMB). The *y*-axis indicates the degree of cortical arousal, which may be distressing dependent on context. The *x*-axis indicates the extent and intensity of NMB. Cortical arousal increases with extent and intensity of NMB, and this is mitigated (antagonized) by increasing depth of anesthesia, as shown by the respective curves. One can imagine multiple curves, more than the 3 shown, each reflecting a specific anesthetic depth. The boxes A–D broadly describe the 4 scenarios described in text and correspond to the movement patterns outlined in Figure 1. Awake patients (red curve) are little distressed with no NMB, but very distressed if fully paralyzed with NMB (box B), especially in context of AAGA. With no NMB, even light anesthesia (box C) is little arousing or distressing. Partial paralysis, or paralysis using IFT (box D) is more arousing than no NMB, but little distressing as patients can move, to some extent. Even with complete global and intense NMB, deep general anesthesia (box A) mitigates arousal or distress.

Globally-partial neuromuscular blockade (vs “regionally-partial” of IFT) has been used widely in physiology research with little or no volunteer distress.^24,25^ Accounts of globally complete, intense paralysis in awake volunteers are rare in the literature but one early report in a single subject by Smith et al. (1947) confirms traumatic feelings of breathlessness, choking and “uneasiness” on emergence.^26^ This is, together with results from AAGA patients in NAP5, consistent with deep NMB being more arousing than any less-than-complete paralysis. These interactions of NMB and depth of anesthesia on arousal are summarized in Figure 2, and to explain how these interactions may arise, we turn to the “efference copy network” in neuroscience.

## GUILLERY’S EFFERENCE COPY NETWORK: HOW MOVEMENT MIGHT CONTRIBUTE TO CONSCIOUSNESS

I attribute the core idea to Guillery,^27–29^ for personal reasons explained in acknowledgements,^30^ but many researchers before and after developed and refined the ideas (see Supplementary Digital Content, https://links.lww.com/AA/F448).^31–34^ Efference copies have been studied across several systems, especially eye and limb movements and cerebellar control. The anatomy of the pathways is well defined, and although granularity is lacking for all motor systems, the principle is well supported by functional and physiological evidence.

The notion of efference copy challenges the common teaching that sensory and motor systems are completely anatomically and functionally separate. In the traditional “sandwich model,” afferent stimuli leading to perception are sent to a sensory receiving area in the neocortex. Separate to this area is the motor cortex. Functionally sandwiched in between is a decision-making or cognitive center that links perception to action. This cognitive center is ill-defined, and amongst other limitations, it has long been recognized that the sandwich model cannot explain how we can immediately distinguish sensations created by our own movements from those imposed upon us by the environment. For example, even simple animals like earthworms withdraw rapidly to touch; yet crawling on a surface, producing identical cutaneous stimulation, evokes no withdrawal. The worm is not rendered immobile by its own attempted movement and can identify, and hence discount, sensations produced by its own motions.^24^ Whichever mechanism facilitates this also explains why, trivially, we cannot tickle ourselves to laughter. The proposed explanation lies in efference copy.

**Figure 3.**
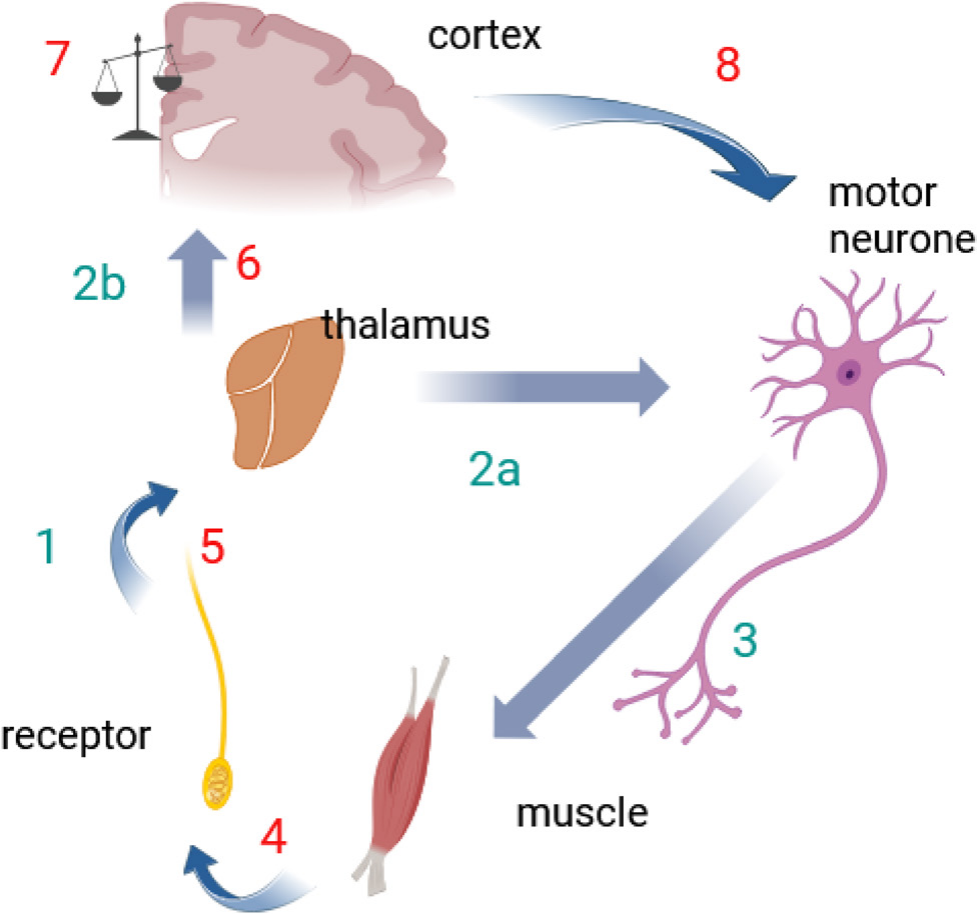
Summary of the efference copy-reafferentation loop, after Guillery and colleagues. In sequence: (1) sensory input relays at thalamus, via branched afferents that synapse with motor efferents and afferent relays. The thalamus as a site is really a summary, because Guillery noted there existed other branches between afferents and motor neurones, in phylogenetically older motor centers in brainstem, cerebellum, striatum and superior colliculus. From here, information is relayed in two directions: (2a) to initiate motor actions and 2(b), to the higher cortical centers, communicating not only the sensory information, but also the motor actions that are already initiated in response to that sensory information in 2(a). This, 2(b), constitutes the efference copy. The motor neurones activated by 2(a) send the onward command to the muscles (3). Reafferentation is indicated in red numbers. The motor actions are in turn detected by receptors (4) that send new sensory information to the cortex (5) and (6). Now, the cortex can compare if what was done by efference copy command is what should have been executed (weighing scale at 7), and if necessary, fine tune or override any further efference copy response (8). However, if it does so, then it becomes aroused and aware of the sensations and actions; and it is from this arousal that Guillery argues that “consciousness arises from our movements.”

Guillery’s suggestions rested on neuroanatomical observations that the afferent sensory pathways enter, and then branch within the phylogenetically “oldest” part of the central nervous system, in the brainstem and spinal cord. Even simple premammalian vertebrates rely on direct connections between these afferents and motor neurones to generate appropriate actions. Guillery also noted the extensive afferent-efferent branching within the thalamus of mammals and proposed that information from this phylogenetically older part of the nervous system was sent back up to the neocortex. That is, a copy of the motor instruction already produced by the lower-center links between afferent and efferent systems (“efference copy”) is sent to the cortex *together with* the original sensory information (Figure 3). This idea was different from some earlier notions of efference copy (Supplemental Digital Content, https://links.lww.com/AA/F448).^27–29^

Guillery considered how reasonably accurate and consistent motor responses could be generated and maintained over an animal’s lifetime, and suggested that the subcortical connections were the simplest for an organism to develop (evolve) and little prone to error. The motor responses that ensue were both hard wired through evolution and soft-wired (learned) during growth and development. He was emphatic: (my emphasis): “When a new sensory receptor is acquired in the course of evolution it will have *no* survival value if it lacks a [preformed efference copy] motor output.”^29^

Efference copy means that the brain (mind) is ‘made aware’ of the sensory input as well as the immediate response already initiated. Guillery uses a military analogy where a general [neocortex] receives a message from a battlefield commander [thalamic relay] to the effect that “the enemy is seen at the river [sensory input]; a squadron has been sent to the bridge [efference copy response].”^29^

This network readily enables us to distinguish sensations created by our own movement and those imposed on us by the environment through a third aspect of the network: the principle of “reafference.” The executed motor actions resulting from the efference copy to the muscles described above itself result in afferent sensory signals (touch, proprioception, etc), that provide information about the effectiveness of that set of movements, and are, in informatic terms, associated with them. Guillery argued that through these ‘reafferents’, the brain can assess whether the efference copy command has had the appropriate effect. To extend the military analogy above: “did the squadron that was sent, remove the enemy?” If the information matches and the efference copy response is appropriate (the enemy was dislodged), the neocortex needs take no further action: it can remain in a “relaxed” state and need not have any real perception/awareness of what has happened; the lower centers have done all the work. Thus, the majority of our actions are indeed “automatic” in this way and we are not consciously aware of them.

If, however, there is a perceived shortcoming in the motor response—a mismatch—then further cortically-led adjustment to motor instructions may follow. The higher centers are then activated (aroused) as they need to do some work and send further corrective motor commands. The brain here faces uncertainty, which it needs to resolve, as to whether the mismatch arose due to some rapid change in the external environment, or because of error in the original efference copy-driven movement. Resolving this uncertainty and sending fresh commands arouses the neocortex. In the military analogy, perhaps the squadron did not follow the command to move to the bridge, or having got there, did not dislodge the enemy; the general now needs to be alert and take action.

Guillery went further in radically linking all this to our conscious awareness of the environment.^27–29,35,36^ He noted that the classical sandwich model did not really explain how action and perception reciprocally relate to each other. For example, in searching for a small coin in our pocket, he argued that we are not focused on our movements, yet it is movement that creates the perception (eg shape, size, weight) of the coin. This association of sensory and motor functions meant to him and his colleagues that ‘consciousness’ did not solely cause movement (as in the traditional sandwich model). Rather, and somewhat boldly, he asserted that consciousness resulted from our motor actions, especially with cortical arousal in the face of reafferent/efference copy mismatch.

This notion was independently suggested by Tversky^37^ and others^38^ in the realm of cognitive science, where they proposed movement as the foundation of thought. These ideas together emphasized that movement control is a very low-level function of all animals. Movement beyond the most basic reflexes requires some form of planning and so might be intimately connected to other cognitive apparatus.

## EXPLAINING MOTOR RESPONSES UNDER ANESTHESIA BY REFERENCE TO EFFERENCE COPY

If efference copies are central both to motor responses and to consciousness in this way, then they might explain the discrete patterns of responses outlined in Figure 1 in the manner described below; notwithstanding the special case that during deep anesthesia (scenario A) the efference copy network is suitably silenced, along with other brain functions, and there is no movement to stimuli.

However, at lighter planes of anesthesia with complete NMB paralysis (scenario B, Fig. 1), the efference copy mechanism is partly active. Sensory inputs from surgical stimulation can evoke a subcortical signal to move, along with an efference copy. However, NMB prevents movement of any part of the body. Reafferentation thus indicates a severe, global mismatch, which arouses the cortex. Ever-stronger cortical signals to move fail to produce a response, and the cumulative effect results in cortical awakening and psychologically traumatic AAGA (Supplementary Digital Content, https://links.lww.com/AA/F448).

In an alternative situation, at equivalently light planes of anesthesia but with no NMB (scenario C, Figure 1), the efference copy is also partly active. Sensory inputs from surgical stimulation evoke an albeit unsophisticated (anesthesia-blunted) subcortical signal to move, along with an efference copy. Movement is here possible (limb jerks or facial grimacing, etc), so the reafferentation information is interpreted as “appropriate” by the anesthesia-blunted neocortex. The higher brain centers remain “calm” and there is no further arousal into AAGA. In practice, the anesthesiologist observing this movement will deepen anesthesia but if they fail to do so, then stronger afferent signals with surgical stimulation could result in a reafferentation mismatch and AAGA—although, as NAP5 data indicate, this is very rare at 1 in 135,000 cases.^1,2^

We can now turn to scenario D (Figure 1), the IFT construct, where anesthesia is also light. As in the scenarios above, the efference copy mechanism is partly active and sensory inputs from surgical stimulation evoke an albeit unsophisticated subcortical signal to move, along with an efference copy. Here almost all the muscle groups receiving the diffuse commands to move are NMB-paralyzed so the degree of mismatch perceived by the brain through reafferentation, and hence initial degree of cortical arousal, is similar to scenario A, with complete NMB. However, in IFT there are frequent verbal commands given: this sensory input results in a meaningful movement response to those commands from this greater-aroused neocortex. Moreover, this movement mitigates against any further cortical arousal to full awareness, as it is interpreted as a “matched” response to stimulus, within the reafferentation/efference copy network. The brain is not further aroused into traumatic AAGA.

The reason why spontaneous movement with surgical stimulus during IFT is not so frequently seen, as it is in scenario C, is simply that the IFT is an experimental construct in which so many verbal commands are given at repeated intervals, such that any spontaneous movement can only arise in the relatively brief interludes between commands. If patients using the IFT construct were ever left undisturbed, it is likely that occasional spontaneous movement would be seen. In a systematic review^5^ that catogorised IFT movements based on an original scale suggested by Wilson,^39^ IFT patients did occasionally (11%) show random jerks as a type of movement, but not always contiguous with surgical stimulus. Note that the normal response of the anesthesiologist to a response to verbal command in IFT is correctly to deepen anesthesia. If they failed to do this, there could indeed be full awareness and potential AAGA, by the same network mechanisms outlined above. Consistent with this, Tunstall described keeping a patient largely awake but paralyzed, and “conversing” with her using IFT throughout surgery.^40^ Her ability to move and continuing verbal reassurance presumably prevented any psychological trauma. Tunstall’s “experiment” would not be now considered a wise or even ethical approach.

## LIMITATIONS TO THE EFFERENCE COPY THEORY AS APPLIED TO MOVEMENTS DURING ANESTHESIA

In some additional support for the theory, we can consider the contrast between neuraxial analgesia and neuromuscular blockade. In the former, the total blockade of efferents and afferents means that there is no afferent/reafferentation signal for comparison; i.e, no mismatch. The cortex remains calm and patients are comfortable and somewhat sedated.^15^ With NMB however, both efferent and afferent neurones are active, creating potential for mismatch, and hence the cortical arousal or AAGA described above. However, while the efference copy network offers a novel explanation for this example and the patterns of response in Figure 1, the theory has its limitations. One is that the proposition could be criticized as being merely a *post hoc* ‘theory of convenience’, and does not itself lead to a testable prediction. Expressed differently, even intimate knowledge of the efference copy network did not lead anyone hitherto to link it to anesthetic mechanisms and *predict* the IFT and the other discrete responses summarized in Figure 1. Generally, theories of anesthesia mechanisms have focused on drug interactions with brain sleep and arousal networks.^41^ So considerable refinement and discussion will be required within the anesthesia community, for the efference copy network to enter into our natural language.

Another limitation is that the patterns of movement response on which the theory rests, are not necessarily as clear-cut as implied by Figure 1. The suggestions that partial NMB (or the “regionally-partial” NMB of IFT) is more arousing than no NMB, and that complete, intense NMB is more arousing than partial or IFT all discount the possibility that differences between these conditions may equally well be due to the priming of volunteers in the partial NMB experiments, and the prior consent and preparation of patients in the IFT studies. The proposition also relies heavily on the ‘paradoxical’ observations that (a) patients in the IFT construct (scenario D) *only* respond to verbal command and are not witnessed to move spontaneously to surgery; and (b) that patients under anesthesia with no NMB (scenario C) *never* respond with movement to verbal command.^11,12^ If somebody reports that in fact, the pattern of response in these two scenarios is identical, this would potentially undermine explanations based on the efference copy network. Equally undermining would be if separate neuroscientific research questioned the efference copy network as integral to brain function. For example, Guillery’s emphasis on efference copies being a basis for consciousness remains controversial.^42^ That all said, one important aspect of the argument is clear: neuromuscular blockade increases cortical arousal, in proportion to the intensity of NMB (Figure 2).

The brain’s capacity to integrate information is thought important in explaining anesthesia mechanisms, and an example is ‘information integration theory’ (IIT), with Tononi proposing a complex metric (φ) based on entropy.^43^ The proposition based on efference copy network is not incompatible with this, as Guillery’s approach relies on information being integrated first, at the level of the thalamus through the branching networks linking sensory afferents to motor neurones (step 2b in Figure 3) and second, in the higher centers comparing reafferentation feedback with the executed motor command (step 7 in Figure 3). Moreover in Guillery’s theory, the greater uncertainty—or entropy—there is (ie, mismatch between what was intended and the reafferent signal), the higher is the level of arousal/consciousness. Recently, the EEG phenomenon of slow wave saturation (SWAS), appearing at the point of clinical loss of consciousness, has been proposed to reflect thalamic isolation wherein the thalamocortical system becomes isolated from sensory stimuli. This is also consistent with the notion that efference copy signals can be inhibited at this point from being sent to higher centers.^44^ It is beyond the scope of this article to relate the literature around IIT and SWAS comprehensively to the efference copy network, but these examples illustrate some areas for future discussion.

## CONCLUSIONS

In summary, stimulation of peripheral receptors evokes subcortical motor responses and activates the efference copy network. If the resulting motor response is thwarted, as in neuromuscular blockade, the neocortex is aroused; with inadequate anesthesia sometimes to the point of distressing AAGA. The ability to move, as facilitated by partial neuromuscular blockade or the IFT construct, is like a “release of tension” and cortical arousal is mitigated. Because it is most of the body that is paralyzed in IFT, the state of cortical arousal is higher than it is with no NMB, and thus IFT patients exhibit a higher level of cognitive capacity in being able to respond to verbal command with their unparalysed forearm (which patients with no NMB do not), despite being in an equivalently light plane of anesthesia.

By introducing the anesthesia community to the efference copy network and its involvement in movement and conscious perception, it is hoped to stimulate debate in new directions about alternative mechanisms for how anesthetics might induce unconsciousness and/or unresponsiveness.

## ACKNOWLEDGMENTS

I am indebted to conversations with the late Professor Rainer Walter (Ray) Guillery (1929–2017), who as Dr Lee’s Professor of Anatomy at Oxford, UK (1984–1996), taught me neurophysiology and neuroanatomy. I am grateful to Professors Andrew Parker and Armin Lak, Professors of Neuroscience at the University of Oxford, UK, for helpful comments on this article. Figures 2 and 3 were created with the assistance of Biorender (licence agreement numbers YY283O9H4Q and YA283O9TGB, respectively).

## DISCLOSURES

**Conflicts of Interest:** J. J. Pandit is Editor-in-Chief, *Anesthesia & Analgesia*, and was not involved in the handling of this article. **Funding:** None. **This manuscript was handled by:** Jiro Kurata, MD, PhD.

## Supplementary Material
