# Supplementary material for: Movements During Intended General Anesthesia and Psychologically Traumatic Accidental Awareness: Explanatory Role of the “Efference Copy Network”
Source: Anesth Analg. 2025 Sep 5;142(5):909–16. doi: 10.1213/ANE.0000000000007722 (PMC13048314; doi:10.1213/ANE.0000000000007722)
Supplement: Supplementary file 1 [file ane-142-909-s001.pdf]

Supplementary digital content

Online Supplement S1. Brief outline of efference copy

Arguably the first to entertain a notion of efference copy was von Helmholtz (1866); a German physician and physicist. He noted that whereas the visual world is stable with active head or eye movement, pressing on one's own eye causes the visual world to move. He reasoned that there must be some mechanism linking action to perception in a way that enabled the mind to distinguish between sensations created by the body's own movement. With the passive movement of the eyeball, no efference copy is generated (as they are with active movements) so no sensory changes are anticipated and discounted; thus the visual appears to move.

Thus, original literature on efference copy envisaged it as originating from the higher centers, and being a copy of the information these centers sent to the muscles (Grüsser, 1995). This efference copy provided input to a conceptual 'internal model', predicting the sensory feedback that would arise from the original motor command. The actual sensory consequences arising from the muscle actions are then compared with these predicted, to inform the higher centers about how well the two (predicted and actual) are matched.

The model of Guillery and colleagues is decidedly different, as discussed in the main body of text. For them, the efference copy originates within the more direct branching and synapses of sensory afferents with motor neurones within the thalamus. The efference copy is information sent to the higher centers from these lower centres of a motor command that has already been given. Other elements of Gullery's model are similar to previous models, namely the re-afferent limb and the comparison of what was intended with what actually occurred. Gulliery's approach more readily fits with his and others' proposal that consciousness *arises from* motor actions and that these are not just the result of consciousness.

However, both the notion of efference copy and specifically, Guillery's model are not without critics. Sherrington criticised efference copies as long ago as the early 20th century, favouring instead the notion that muscle afferents, including proprioceptors were sufficient to provide the required information to guide action (Matthews, 1982). Another alternative idea is that of 'referents'; i.e., that the brain has a reference for a stable position and any movements that deviate from this are compared, and if necessary corrected (Feldman, 2016). Separately, Guillery's proposal of consciousness arising from motor action has been questioned on philosophical grounds by, amongst others, Hacker, who criticised it as part of what he called the

‘mereological fallacy’; the tendency by neuroscientists to ascribe to the brain psychological concepts that only make sense when ascribed to whole animals (Schaal, 2005; Bennett and Hacker, 2024).

Online Supplement S2. Volunteer reports supporting the notion that attempts to move when paralysed arouse higher cortical centers

Schuller et al. (2023) offer volunteer reports of awake paralysis indicating that NMB causes arousal, and moreover, distress where attempts to move are made in the face of paralysis. The text is as follows:

“Some [volunteers] did not even realize that they were fully paralyzed until they deliberately tried to move.”

“If subjects tried to breathe during paralysis, it immediately invoked a sense of suffocation and panic...”

“Attempts to swallow induced a sense of suffocation in four subjects...*’I honestly felt that I was going to die’*.”

“During dense paralysis, however, four subjects reported that any attempt to move a limb led to an immediate “horrible” sensation, which did not appear to be localized to the limb and which had a strong affective component. It was striking both in its unpleasantness, and its rapidity of onset, and it vanished almost as quickly once the attempted movement was ceased. Subjects were surprised to find they disliked the sensation so much they were not keen to try it again.”

And the mitigation of distress offered by the isolated forearm technique (IFT) is reported as:

“The importance of the isolated forearm cannot be overstated. *‘Communicating with my hand was very comforting. I felt very warm toward the person holding my hand. When they let go, you don’t know if they are there anymore. It’s very lonely without that person holding your hand.’* Another said, *‘The isolated forearm seemed to embody my only existence on earth. I felt as if I was hanging on to the outside world with that hand.’* “

Patient reports from the NAP5 study also indicate that attempts to move in the face of paralysis are increasingly arousing to the point of distress (NAP5, 2015):

“The patient wanted to scream but could not move or speak. The patient developed nightmares, waking up crying in a cold sweat recalling events repeatedly. The patient described feeling imprisoned in their own body.”

“The patient tried to move but was unable to and was terrified of “enduring the torment”. The patient experienced flashbacks, re-living experiences and felt traumatised.”

“The patient could not move. The patient remembered trying to cry in an attempt to alert the anaesthetist…The patient is scared of future anaesthesia.”

“The patient knew something was wrong and panicked because they could not move . The patient tried to scream out but could not. The patient “…felt violated”

“The patient remembered being unable to move, breathe or speak and feared death. The patient developed flashbacks..”

“The patient reported inability to move and that they were trying to move…The patient suffered psychological distress…”

“The patient tried to shout and move, but could not and…subsequently developed flashbacks and re-living experiences, and felt traumatised.”

“The patient felt paralysed, unable to speak or move and feared death. The patient became terrified about future anaesthetics, with sleep disturbance and worry about death on a daily basis.”

“She was distressed and felt ‘terrified’, tried to blink and move her arm to alert people but was unable to do so.”

Bibliography to Supplement

Bennett MR, Hacker PMS. The Representational Fallacy in Neuroscience and Psychology: A Critical Analysis. Palgrave Macmillan: 2024.

Feldman AG. Active sensing without efference copy: referent control of perception. J Neurophysiol. 2016; 116: 960-76.

Grüsser OJ. On the history of the ideas of efference copy and reafference. Clio Med. 1995;33:35-55.

Helmholtz H. Handbuch der Physiologischen Optik. Leipzig: Voss, 1866

Matthews PB. Where does Sherrington's "muscular sense" originate? Muscles, joints, corollary discharges? Annu Rev Neurosci. 1982; 5:189-218.

NAP5, 2015 At: https://www.rcoa.ac.uk/research/research-projects/national-audit-projects-naps/nap5-accidental-awareness-during-general

Schaal DW. Naming our concerns about neuroscience: a review of Bennett and Hacker's philosophical foundations of neuroscience. J Exp Anal Behav. 2005; 84: 683-92.

Schuller PJ, Voss LJ, Barry JJ. Awake total neuromuscular blockade as experienced by anesthesiologist volunteers. Anesthesiology. 2024; 140: 336-338.
